# The Art Amateur

**Published:** 1894-09

**Authors:** 


### BOOK NOTICES.

THE ART AMATEUR, August number, has just been received. It is published by Montague Marks, No. 23 Union Square, New York. Price, $4.00 a year; 35 cents a number.

The frontispiece is a portrait of Kossuth, engraved by Gilardi. The section called "Gallery and Studio" contains eleven illustrated articles. The section devoted to china painting contains: Vacation notes for china decorators; Decorative initials for china painting; Landscape and game painting, "Pike;" fourth plate of a "Fish" service of fifteen pieces. The painting of fish. The section on "House" has: Painting on textiles for screens; Carved and painted screen; Talks about embroidery; Portion of an altar frontal; Some English wooden mantlepieces; Present wall-papers and other hangings, and English and American decorations.

It treats also of new publications. The supplement designs contain decoration for a bonbonnière; "Red-head Ducks," sixth of a set of twelve game plates; Beau motive for embroidery; Vase decorations; Decoration for a fan, or cover for a blotter; Initial letters for embroidery; Border for embroidery; Butterfly decoration for painting or pyrography, and nasturtium decoration for a calendar. The color-plates are two: "The Lightship" and "Butterflies."

This number is as valuable as its predecessors, and we welcome it.

